# Sex and age differences in the expression of liver microRNAs during the life span of F344 rats

**DOI:** 10.1186/s13293-017-0127-9

**Published:** 2017-02-03

**Authors:** Joshua C. Kwekel, Vikrant Vijay, Tao Han, Carrie L. Moland, Varsha G. Desai, James C. Fuscoe

**Affiliations:** 1 Division of Systems Biology, National Center for Toxicological Research, US Food and Drug Administration, Jefferson, AR USA; 2 Present address: Department of Math & Science, Central Baptist College, Conway, AR USA

**Keywords:** Sex difference, Age difference, Liver, miRNA expression

## Abstract

**Background:**

Physiological factors such as age and sex have been shown to be risk factors for adverse effects in the liver, including liver diseases and drug-induced liver injury. Previously, we have reported age- and sex-related significant differences in hepatic basal gene expression in rats during the life span that may be related to susceptibility to such adverse effects. However, the underlying mechanisms of the gene expression changes were not fully understood. In recent years, increasing evidence for epigenetic mechanisms of gene regulation has fueled interest in the role of microRNAs (miRNAs) in toxicogenomics and biomarker discovery. We therefore proposed that significant age and sex differences exist in baseline liver miRNA expression, and that comprehensive profiling of miRNAs will provide insights into the epigenetic regulation of gene expression in rat liver.

**Methods:**

To address this, liver tissues from male and female F344 rats were examined at 2, 5, 6, 8, 15, 21, 52, 78, and 104 weeks of age for the expression of 677 unique miRNAs. Following data processing, predictive pathway analysis was performed on selected miRNAs that exhibited prominent age and/or sex differences in expression.

**Results:**

Of the 314 miRNAs found to be expressed, 214 were differentially expressed; 65 and 212 miRNAs showed significant (false discovery rate (FDR) <5% and ≥1.5-fold change) sex- and age-related differences in expression, respectively. Thirty-eight miRNAs showed 2-week-specific expression, of which 31 miRNAs were found to be encoded within the Dlk1-Dio3 cluster located on chromosome 6. This cluster has been associated with tissue proliferation and differentiation, and liver energy homeostasis in postnatal development. Predictive pathway analysis linked sex-biased miRNA expression with sexually dimorphic molecular functions and toxicological functions that may reflect sex differences in hepatic physiology and disease. The expression of miRNAs (miR-18a, miR-99a, and miR-203, miR-451) was also found to associate with specific sexually dimorphic hepatic histopathology. The expression of miRNAs involved in regulating cell death, cell proliferation, and cell cycle was found to change as the rats matured from adult to old age.

**Conclusions:**

Overall, significant age- and sex-related differences in liver miRNA expression were identified and linked to histopathological findings and predicted functional pathways that may underlie susceptibilities to liver toxicity and disease.

**Electronic supplementary material:**

The online version of this article (doi:10.1186/s13293-017-0127-9) contains supplementary material, which is available to authorized users.

## Background

The liver plays a crucial role in metabolizing food and drugs, and thus is of great toxicological importance. Physiological factors such as age and sex have been suggested as risk factors for liver diseases and drug-induced liver injury (DILI). For example, anti-epileptic drugs, such as carbamazepine and valproate, have been associated with age-dependent susceptibilities to adverse effects [1, 2]. Elderly patients who take carbamazepine showed an increased risk for adverse liver reactions [3, 4], whereas younger patients taking valproate show increased risk for hepatotoxicity [5]. In addition, other drugs, including erythromycin, halothane, isoniazid, nitrofurantoin, and flucloxacillin, have recently been associated with age-dependent increases in adverse liver effects [6].

Moreover, sex-related differences in susceptibility to DILI have been observed. For example, female patients show increased incidence of anesthesia-related DILI compared to males [7]. This sex-bias for anesthesia-related DILI was also demonstrated in a mouse model, which was further linked to an estrogen-dependent pro-inflammatory response involving IL-6 suppression of regulatory T-cells [8]. Clinical and epidemiological studies suggest that females show more sensitivity to adverse events after taking acetaminophen [9, 10], and evidence supporting a general female-bias in DILI has recently been reviewed [11]. However, a precise mechanism for the sex difference is not clearly understood. Several other drugs exhibit sex-biased sensitivities to DILI, but it is not clear whether males or females show more sensitivity to DILI in general [12–14], suggesting the sex bias may be drug-dependent. Establishment of the underlying molecular basis for such age- and sex-related susceptibilities to toxicities or diseases will enable safer use of such drugs.

We have previously reported marked age- and sex-related differences in basal hepatic gene expression in rats [15]. Differential expression was observed for over 3500 genes in a variety of different pathways. It is recognized that messenger RNA (mRNA) expression is under the control of various transcriptional and post-transcriptional mechanisms. miRNAs are short, non-coding RNAs of 19–23 nucleotides in length that regulate gene expression, typically by translational repression of target mRNAs [16–19]. A single miRNA may target a variety of mRNA transcripts and single transcripts may be targeted by multiple miRNAs; thus, their role in mRNA regulatory networks is complex. miRNAs have been shown to influence diverse biological pathways and functions, with a prominent role in various cancers [20, 21]. Furthermore, miRNA biomarkers have shown promising utility in distinguishing cancer types [22], detecting various tissue injuries [23, 24], and making predictions regarding disease outcomes [25]. Altered miRNA expression has been reported in response to liver diseases and injuries, including hepatitis C infection [26], non-alcoholic fatty liver disease (NAFLD) [27], and DILI [28, 29]. Recent studies suggest that miRNAs may play an important role as biomarkers and tools in toxicogenomics [30–32].

A few miRNAs have been shown to exhibit age- [33–35] and sex-dependent [36] patterns of expression. miR-34a was among the first miRNAs to be characterized for its role in aging and longevity in *C. elegans* [37, 38]. miR-34a is a downstream target of p53 and its expression correlates positively with age [39]. Similarly, increased liver expression of miR-93, miR-214, and miR-669c has been correlated with age. These miRNAs target glutathione-S-transferases, which are crucial in protecting against DILI-related oxidative stress [40]. Li et al. [33] have also shown age-dependent increases in the expression of miR-34a and miR-93, and that these miRNAs target Mgst1, Sirt1, and Nrf2, three genes that encode proteins important in the defense against oxidative stress, a common feature in DILI. These studies demonstrate the influence of miRNAs on pathways related to DILI in an age-dependent manner and thus may help develop a fuller understanding of age-related susceptibilities to drug toxicities.

The roles of miRNAs in sexually dimorphic physiology and diseases have recently been reviewed [36]. Sex differences in expression can be regulated through both genetic factors (e.g., X-chromosome encoded miRNAs) and hormonal factors (e.g., sex steroids) [41]. The primary direct regulator of sex differences in gene expression in young adult and adult liver is growth hormone [42]. In a carbon tetrachloride-induced liver fibrosis mouse model, females exhibited protection against fibrosis compared to males, which was associated with miR-29 family members. The miR-29 family members are induced by estrogen and reduce fibrosis by inhibiting expression of collagens [43]. Additionally, male-biased expression of miR-216a via the androgen pathway has been reported in early hepatocarcinogenesis [44]. miR-216a has been shown to target and decrease levels of Tslc1, whose expression is decreased in a number of tumors [45], thus implicating the sex-biased expression of miR-216a in carcinogenesis. Taken together, there is evidence to suggest potential roles for miRNAs in age- and sex-related susceptibilities to liver diseases and toxicities.

Currently, no comprehensive reports characterizing sex and age differences in miRNA expression in rat liver tissue are available. In the present study, whole genome expression profiles of miRNA in the liver of male and female F344 rats during the life span were measured. Results showed substantial sex and age differences in basal liver miRNA expression levels that may have a role in regulating expression of genes associated with hepatic proliferation and differentiation, fibrosis, and steatosis. These data will facilitate a better understanding of epigenetic regulation of sex- and age-related differences in hepatic gene expression and will help elucidate the molecular basis underlying differential susceptibilities to toxicities at different stages of life and between the sexes.

## Methods

### Animal study

All tissue samples came from a previously described study [15] and were maintained at −80 °C prior to utilization in the present project. Briefly, F344 rats were obtained from the National Center for Toxicological Research (NCTR) rat breeding colony. Male and female animals were euthanized at 2, 5, 6, 8, 15, 21, 52, 78, and 104 weeks of age as previously described. Female estrus cycles were not evaluated or synchronized. Animal care and procedures were approved by the NCTR Institutional Animal Care and Use Committee. Animals were housed in polycarbonate cages with hardwood chip bedding, two per cage, at 23 °C with a relative humidity of approximately 50%. Rats were euthanized at the same circadian time of day (between 0900 and 1100) by carbon dioxide asphyxiation. Liver tissue sections from rats aged 52, 78, and 104 weeks of age were collected for histological evaluation (Fig. 7). Tissue sections were placed in 10% neutral buffered formalin followed by standard paraffin embedding, sectioning, and hematoxylin and eosin staining. Liver sections were evaluated for lesions, including basophilic foci, hemorrhage, hyperplasia of the bile duct, hepatocyte hypertrophy, lymphocyte infiltration, chronic active inflammation, necrosis, portal vein thrombosis, and hepatocyte cytoplasmic vacuolization by staff board-certified pathologists. Each lesion was graded for severity using the following scale: 1 = minimal, 2 = mild, 3 = moderate, and 4 = marked. Liver sections were not examined in the younger rats because lesions are only generally found in older rats [46]. Contemporaneous National Toxicology Program studies, using F344 rats from the same source and living in the same environmental conditions at NCTR (including feed) found no basophilic foci, hyperplasia of the bile duct, or hepatocyte cytoplasmic vacuolization in rats of 6–7 weeks of age (0/12 males and 0/12 females) and rats of 17–18 weeks of age (0/24 males and 0/24 females) [47].

**Fig. 1:**
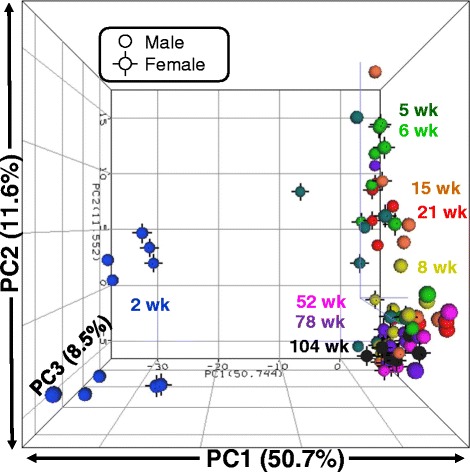
PCA plot of differentially expressed miRNA profiles of 88 rat liver samples. Normalized log2 intensity values for 214 miRNAs that were differentially expressed by age and/or sex in livers of rats of nine ages and both sexes are plotted. Each *sphere* represents the composite DEM profile from a single animal. *Colors* represent age groups. *Black vertices* indicate female animals; males have none

**Fig. 2:**
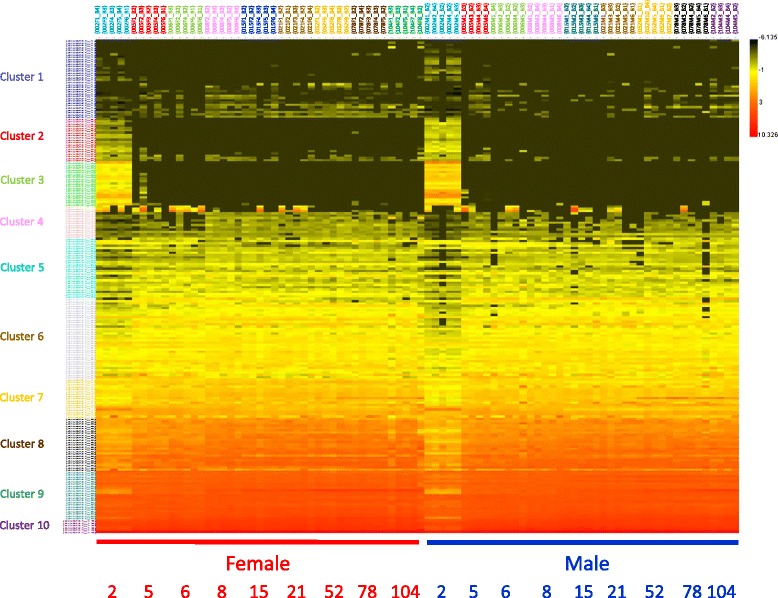
Heatmap of *k*-means cluster analysis of 214 differentially expressed miRNAs in 88 rat liver samples. Normalized log2 intensity values for 214 miRNAs that were differentially expressed by age and/or sex in liver of rats of nine ages and both sexes were used for *k*-means cluster analysis where *k* = 10 clusters. Intensity scale is shown on the upper right with *red* indicating high expression and *black* indicating low expression. Clusters are identified on the *left* and miRNAs identified in Additional file 2. Age (in weeks) is along *bottom*.

**Fig. 3:**
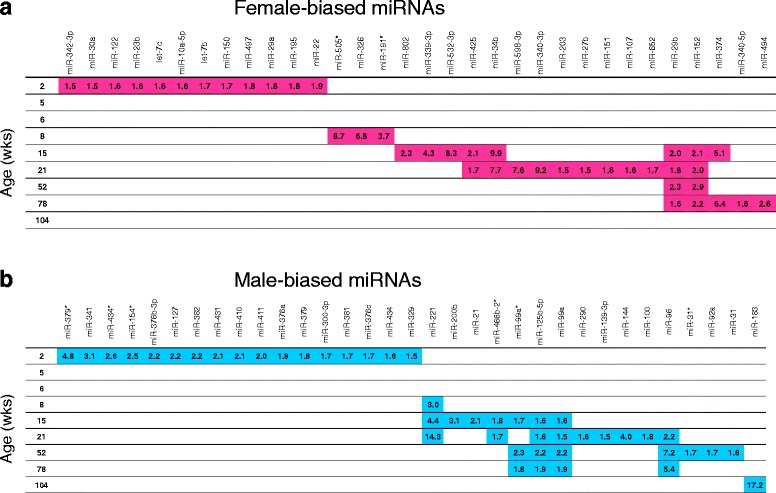
Sex-biased miRNA expression at nine ages in the livers of rats. Sex-biased miRNAs are displayed along the *horizontal axis* for females (**a**) and males (**b**). Age of the rats is shown on the *vertical axis. Numbers* indicate fold-difference in expression of the indicated miRNA, female/male (**a**) and male/female (**b**).

**Fig. 4:**
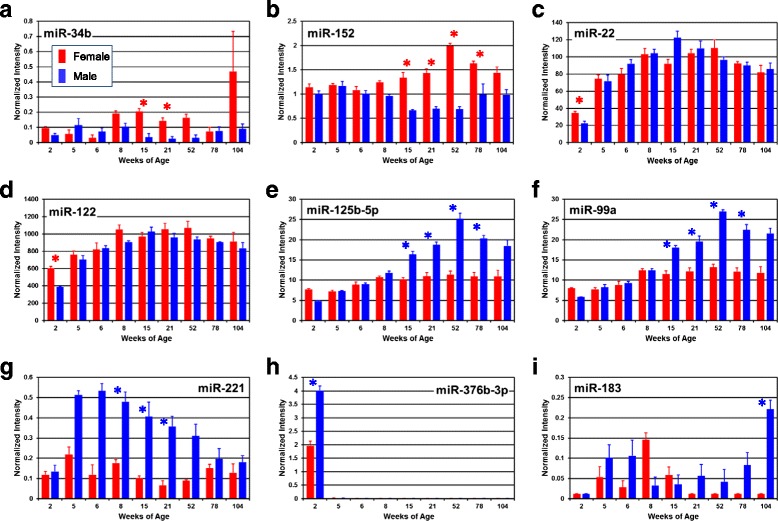
Individual miRNA expression profiles. The expressions of DEMs exhibiting significant differential expression by sex and/or age are plotted (**a–i**). *Y-axis* represents normalized intensity level; *X-axis* represents age in weeks. *Red* and *blue* bars represent female and male group averages, respectively, with standard errors of the means shown. An *asterisk* indicates ages at which a significant sex difference (≥1.5-fold change and *t* test with FDR <5%) is present

**Fig. 5:**
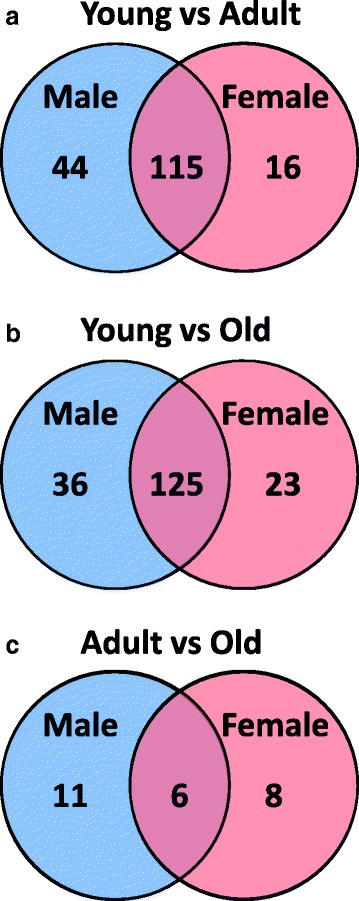
Age differences in expression of miRNAs. Venn diagrams showing the number of differentially expressed hepatic miRNAs between different age groups of male (M) and female (F) rats. **a** Differences and commonalities between young (Y; 2 weeks of age) and adult (A; 5–21 weeks of age). **b** Differences and commonalities between young (Y) and old (O; 52–104 weeks of age). **c** Differences and commonalities between adult (A) and old (O)

**Fig. 6:**
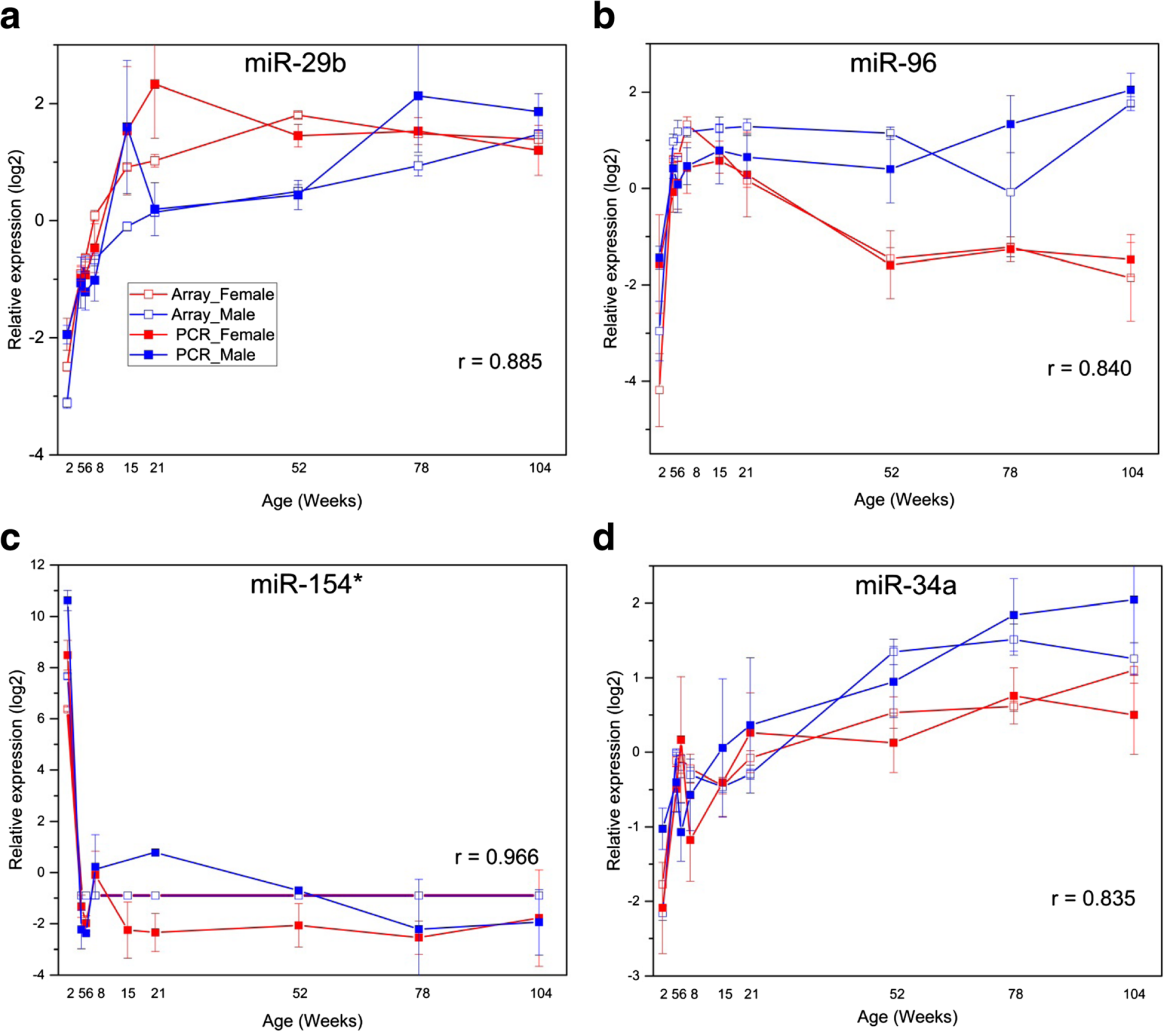
Quantitative PCR verification of microarray results. The expression of select miRNAs was evaluated by Taqman quantitative PCR (qPCR) (**a–d**). The *y-axis* represents relative miRNA expression; the x-axis represents age of rat when euthanized. The *red* and *blue lines* represent female and male, respectively (■: PCR; □: microarray). Standard errors of the means are shown at each age except for MiR-154* (panel **c**) where it was undetectable in some assays. The Pearson correlation coefficient (*R*) is listed in the figure to show the concordance between the two methods

**Fig. 7:**
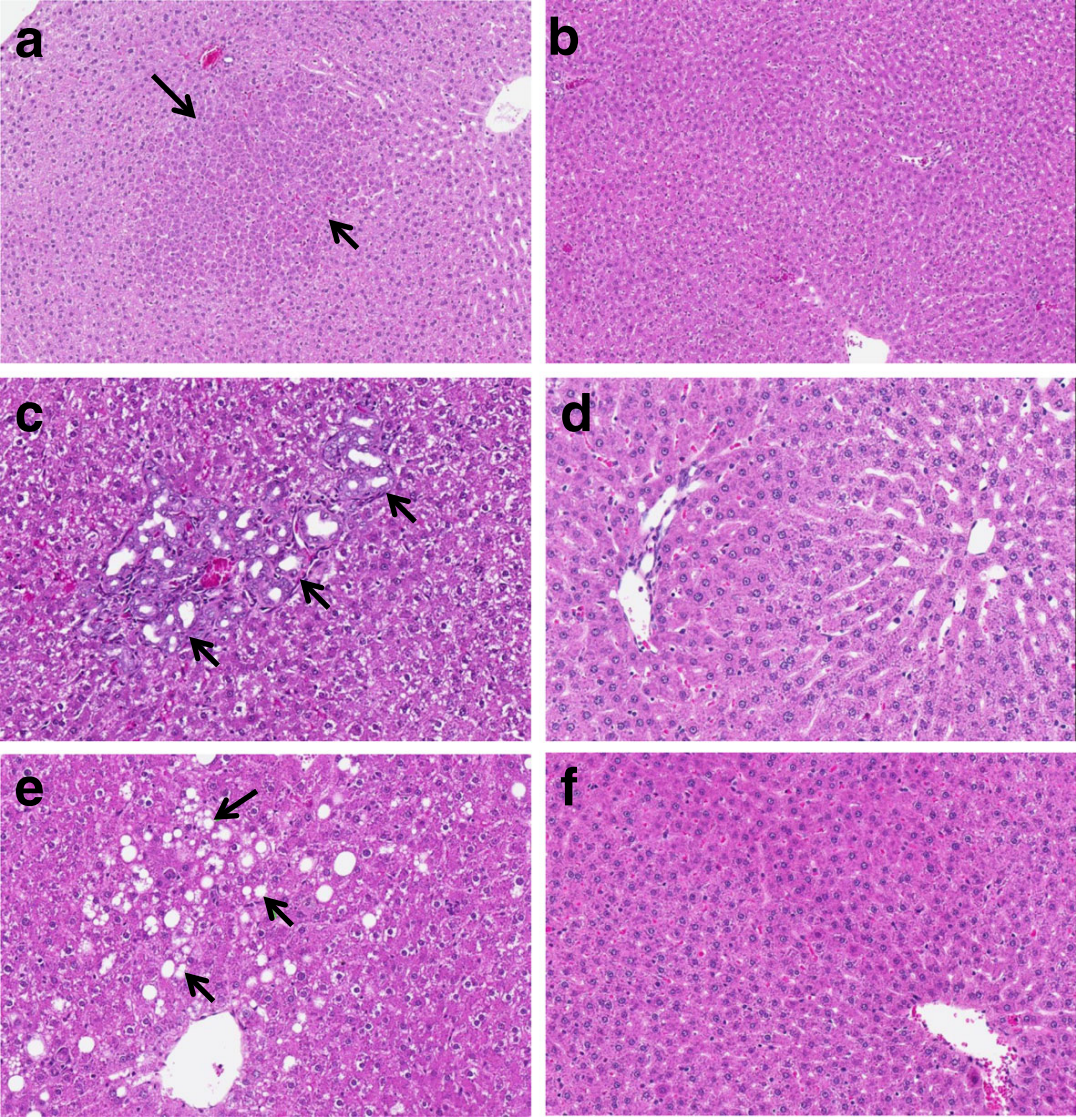
Histopathological assessment of sex-biased hepatic lesions. Liver sections (hematoxylin and eosin stained) from male and female rats at 52, 78, and 104 weeks of age were analyzed for the presence and/or severity of basophilic foci, hyperplasia of the bile duct, and vacuolization of cytoplasm in hepatocytes. Representative images are shown of (**a**) female-biased basophilic focus from a 104-week-old female rat and (**b**) normal liver from a similar area of a 104-week-old male rat; (**c**) male-biased hyperplasia of the bile duct (minimal) from a 52-week-old male rat and (**d**) normal liver from a similar area of a 52-week-old female rat; (**e**) male-biased vacuolization of cytoplasm (mild) in hepatocytes from a 52-week-old male rat and (**f**) normal liver from a similar area of a 52-week-old female rat. *Arrows* point to histopathological features

### RNA isolation

Total RNA including the small RNA fraction were isolated from ~30 mg frozen liver tissue that was ground to a powder in liquid nitrogen and homogenized as previously described [15] following the manufacturer’s protocol (Qiagen miRNeasy Mini Kit, Qiagen Inc. Valencia, CA, USA). RNA samples were evaluated spectrophotometrically on a Nanodrop-1000 (ThermoFisher Scientific, Waltham, MA, USA) by measuring the optical density at 260 nm. RNA quality of each sample was subsequently evaluated on RNA 6000 LabChips using the Agilent 2100 Bioanalyzer (Agilent Technologies, Palo Alto, CA, USA). RNA samples with RNA integrity numbers (RINs) greater than 8.0 were used in miRNA microarray experiments; the average RIN was 8.6.

### Microarray experiments

Comprehensive miRNA expression profiling was performed using single color (Cy3) Agilent Rat 8x15k miRNA (version 16) microarrays and labeling reagents according to the manufacturer’s protocol as previously described [34]. A total of 88 arrays were used (*n* = 5 rat liver RNA samples for all groups, except 5- and 104-week-old males, where *n* = 4 rat liver RNA samples). 100 ng of total RNA, including the small RNA fraction, was used as input. Agilent spike-in controls were used to evaluate both labeling and hybridization efficiency, and all were within acceptable ranges. BioRad Micro Bio-Spin 6 columns were used to purify labeled probe (BioRad Life Science Research, Hercules, CA) according to manufacturer’s recommendation. Samples were processed on four separate days (four batches). To reduce possible batch effects, samples from each age-sex group were randomized among the four batches so that samples from each group were processed in three or four of the batches, with the exception of the 8 week old female group which was processed in two batches. Hybridized miRNA microarrays were then scanned on an Agilent DNA Microarray Scanner (Agilent Technologies) according to the manufacturer’s instructions.

### Microarray data processing

The procedure for processing the miRNA microarray data in the current liver data set is identical to the procedure used in the previously published kidney miRNA microarray data set [34]. This procedure and details pertinent to the liver data are described below for reference. Microarray images (TIF) were analyzed using Feature Extraction (FE) software (V10.7) from Agilent for preliminary image quality control evaluations which utilized the labeling and hybridization spike-in probes and features. FE quality control metrics for each slide were within the ranges designated as “good” or “excellent”. Raw median intensities were calculated for each replicate probe (*n* = 20 for each miRNA) in FE and used to generate a single value (Total Gene Signal, TGS) for each miRNA. Individual data files were generated in FE for each array (88 total files). The 88 individual files were combined into a single file containing TGS data for each array. TGS values of 0.1 represent a placeholder indicating miRNA signal below the level of detection for the assay, equivalent to “not expressed”. Values of 0.1 were subsequently replaced by the global minimum intensity value (1.84) across all arrays. A single file containing processed TGS values was used as input for further processing in SAS 9.3 (SAS Institute Inc., Cary, NC). SAS was used to remove control spots and target spot redundancy/replication. The Agilent Rat 8x15k miRNA microarray (v16) contains 15,744 array spots; 2204 control spots were removed, leaving 13,540 spots of target miRNA probes (677 unique miRNAs x 20 replicates per miRNA). Redundant replicates containing identical TGS values were removed for each miRNA, resulting in data for 677 unique miRNAs. The 363 non-expressed miRNAs (miRNAs with original unprocessed TGS values of 0.1 across all arrays) were discarded, leaving 314 unique miRNAs expressed in at least one rat liver sample. The TGS values (raw intensities) of these 314 “expressed” miRNAs were then transformed to log2 values in SAS followed by 75^th^ percentile normalization.

To prevent generating false fold differences from placeholders, transformed 1.84 values were replaced by 0.012 (lowest transformed value among all samples with measured values).

### Microarray statistical analysis

Statistical analysis was performed in SAS using a generalized linear model procedure (proc glm). *Model* (RandommodelANOVA) and *estimate* statements were used to perform ANOVA and pairwise comparisons using least square method to measure the significance of group differences using log2 intensities as the dependent variable and age or sex as independent variables. Significant age differences in miRNA expression were calculated separately for males and females. Significant sex differences in miRNA expression were calculated at each age. The ANOVA *p* value and *p* value from pairwise tests were adjusted to false discovery rate (FDR). The magnitude of group differences was calculated as the difference of average log2 intensities; the anti-log of the resulting value was used as the fold change value between groups. When group absolute fold changes were greater than or equal to 1.5 and FDR for ANOVA as well as pairwise tests were significant (*p* < 0.05), miRNAs were determined to be differentially expressed by age. Differential expression by sex was determined using absolute fold changes greater than or equal to 1.5 between sexes of the same age and a pairwise *t* test with FDR < 5%. Normalized intensity values (normalized TGS) were subsequently uploaded into ArrayTrack [48], the Food and Drug Administration’s relational database for genomic data storage, processing, analysis, and visualization that was created at NCTR [49]. ArrayTrack is available for free [50].

Principal component analysis (PCA) and *k*-means cluster analysis were performed in ArrayTrack on log2-transformed, normalized intensity values.

Raw expression data, as well as processed data, were deposited in Gene Expression Omnibus (Accession ID: GSE65169).

### Taqman qPCR experiments

Quantitative PCR (qPCR) experiments were carried out using Taqman miRNA assays. 5 ng of total RNA from each sample (*n* = 5 per group) was used in a 15-μl reverse transcription reaction using the Taqman miRNA Reverse Transcription Kit (Life Technologies, Grand Island, NY) along with miRNA-specific primers according to manufacturer’s protocol. The reverse transcription product (1.33 μl) was subsequently used in a 20-μl Taqman MicroRNA Assay reaction with predesigned miRNA-specific probes. The qPCR reactions were performed in MicroAmp Optical 384-well reaction plates on the ABI 7900HT real-time PCR detection system (ThermoFisher). Taqman assays were performed for miR-154* (Assay ID: 000478), miR-29b (Assay ID: 000413), miR-96 (Assay ID: 000186), and miR-34a (Assay ID: 000425) due to their age- and/or sex-related expression differences. U6 snRNA (Assay ID: 001973) was used as endogenous control in the Taqman assay and ∆Ct was further normalized to the global average of each miRNA using the ∆∆Ct method. The average log2 relative expression per group of qPCR and microarray data are displayed for miR-29b, miR-34a, miR-96, and miR-154* such that the global average expression for qPCR (*n* = 5) and microarray data (*n* = 4 or 5) is equal to zero. Sex differences in the qPCR data were tested for statistical significance using a simple *t* test (*p* < 0.05). Taqman qPCR and microarray expression profiles for each miRNA were compared using a Pearson correlation coefficient (*r*) in Microsoft Excel.

### miRNA pathway analysis

QIAGEN’s Ingenuity Pathway Analysis software (IPA; QIAGEN, Redwood City, CA) was used to associate differentially expressed miRNAs with their target mRNAs, and pathway analysis. Briefly, lists of differentially expressed miRNAs (using the Agilent probe identifiers) were imported into IPA, and the miRNA target filter function in IPA was used to identify their corresponding mRNA targets. The analysis was restricted to experimentally observed interactions retrieved from TarBase [51], miRecords [52], and curated peer-reviewed literature. The output of this analysis was exported as an Excel file, duplicate mRNA targets removed, and the file of mRNA targets uploaded to IPA. These lists of target mRNAs were then examined for over-representation in biological pathways curated in IPA using the Core Analysis option. All settings were default settings except the species selected was rat. Molecular and cellular functions, as well as physiological system development and function under the diseases and functions tab were identified. Tox functions, including only hepatotoxicity, were examined as well. Pathways and functions were considered significant if there were at least three mRNAs in the pathway/function, and Fisher’s exact test *p* value was less than 10^−3^. The analysis was limited to rat-specific data except for analysis of the 2-week-specific expression class where the more abundant human data was included.

## Results

### Hepatic miRNA profiles change during the rat life span

The microarrays allowed examination of the expression levels of 677 unique miRNAs. More than half (363 miRNAs) showed no detectable expression at any age or sex and were removed from further analysis. The remaining 314 miRNAs showed a detectable expression level in at least one sample. An ANOVA with an FDR <5% was used to identify age-dependent differences in the expression of the 314 expressed miRNAs in males and females, independently. Subsequently, post hoc multiple comparisons tests (*p* < 0.05) with a fold change ≥1.5-fold between age groups, identified 176 and 171 miRNAs that showed differential expression by age in females and males, respectively, for a total of 212 age-affected miRNAs in either sex (Additional file 1). miRNAs showing significant sex differences were identified by comparing males and females at each age using a pairwise *t* test with FDR < 5% and fold change ≥1.5 between the sexes. The application of these criteria identified 65 unique miRNAs that showed sexually dimorphic expression in at least one age. In combination, a total of 214 miRNAs (Additional file 2) were differentially expressed by age and/or sex with 63 miRNAs showing both age and sex differences. These 214 miRNAs were used in further analyses. The expression of more miRNAs was impacted by age than sex, and most sex-related differentially expressed miRNAs (DEMs; 97%; 63/65) also showed an age effect.

PCA, utilizing the expression profiles of these 214 DEMs, was used to evaluate the relationships among the 88 liver samples (Fig. 1). PC1 accounted for a large proportion of the variability (50.7%). Individual animals generally divided into two groups consisting of 2-week-old animals (Fig. 1; blue) and all remaining age groups, suggesting a distinct miRNA expression pattern at 2 weeks of age. The 5 to 104 week age group subdivided slightly between 5 and 21-week-old animals and 52 to 104-week-old animals. Within the 2-week-old group of animals, a slight but consistent division between males (on the left) and females (on the right) was observed. Aside from this slight sexually dimorphic pattern at 2 weeks of age, the remaining age groups showed little to no evidence of sex differences in the PCA plot.

After establishing the global relationships between groups and replicates, *k*-means cluster analysis was used to visualize patterns among the individual miRNA profiles using the 214 DEMs (Fig. 2; cluster members are shown in Additional file 2). An initial number of *k* = 10 clusters was used as an estimate of the number of large-scale expression profiles present within the miRNA data. Three trends in the *k*-means cluster analysis were observed. First, miRNAs generally clustered according to normalized expression level, with highly expressed miRNAs grouping with other highly expressed miRNAs (e.g., clusters 8–10), and low/medium expressed miRNAs with others showing low/medium expression (e.g., clusters 1, 4, 5, and 6). Secondly, miRNAs generally maintained stable expression patterns across age and sex (e.g., clusters 4–10). Thirdly, clusters 2 and 3 diverged from this stable expression profile with a unique 2-week-specific expression pattern in both sexes, followed by little to no expression at subsequent ages. There were a total of 38 miRNAs in clusters 2 and 3 which exhibited a 2-week-specific expression pattern. miRNAs in cluster 3 exhibited the greatest difference in expression between 2 weeks of age and all subsequent ages. Within cluster 3, male and female animals, on average, showed a 28- and 53-fold difference, respectively, in expression between 2 weeks of age and all subsequent ages. Thus, *k*-means cluster analysis helped identify specific miRNAs that account for the prominent age differences observed in the PCA.

### Sex differences in expression of miRNAs

Sex different expression of miRNAs was evaluated at each age (*t* test with FDR < 5% and fold change ≥ 1.5) and shown in Fig. 3a (females) and Fig. 3b (males), along with the expression fold-differences. A total of 32 miRNAs showed significant female-biased expression at any age while 33 miRNAs exhibited male-biased expression. There was no overlap between the 32 female-biased miRNAs and the 33 male-biased miRNAs. While most of the sex-biased miRNAs occurred at just a single age (54 of the 65 sex-biased miRNAs), 11 (5 female and 6 male) were present at more than one age. Female-biased expression of miR-29b and miR-152 occurred from 15 to 78 weeks of age while male-biased expression of miR-125b-5p and miR-99a occurred during this same age span. A single miRNA (miR-183) showed sex-biased expression at 104 weeks of age and was expressed at levels 17.2 times higher in males than females. Twenty-nine miRNAs were expressed in a sex-biased manner in 2-week-old animals (12 female-biased, 17 male-biased), with differences of 1.5- to 4.8-fold (average = 2.0-fold difference). There were fewer miRNAs showing sex-biased expression at subsequent ages: 4 miRNAs at 8 weeks (3 female, 1 male), 15 miRNAs at 15 weeks (8 female, 7 male), 20 miRNAs at 21 weeks (11 female and 9 male), 9 miRNAs at 52 weeks (2 female and 7 male), and 9 at 78 weeks (5 female and 4 male). Animals between 5 and 6 weeks of age showed no significant sex-biased miRNA expression. Thus, most sex-biased miRNA expression occurred at a single age and occurred predominantly at 2 (29 miRNAs), 15 (15 miRNAs), and 21 (20 miRNAs) weeks of age. Persistent sex-biased expression of 4 miRNAs (miRNAs 29b, 152, 125b-5p, and 99a) occurred in adults at 15–78 weeks of age, with expression fold-differences between the sexes of 1.6–2.9. The sex-biased expression of 9 miRNAs in both sexes during the life span is shown in Fig. 4, with panels **a** through **d** showing female-biased miRNA expression, and panels **e** through **i** showing male-biased expression of miRNAs.

### Age differences in expression of miRNAs

Examination of the PCA (Fig. 1) identified a large difference in liver miRNA expression profiles between 2 weeks of age and the other ages along principal component (PC) 1. In addition, there was a smaller, and incomplete, separation of samples along PC2, with 5–21 weeks of age samples generally grouping away from the more tightly clustered samples from the older ages (52–104 weeks of age). Also, as described below, hepatic histopathology was seen in the livers of the older rats (52–104 weeks of age) and not in the livers of the 5–21 week old rats. For these reasons, the 9 age groups were divided into 3 age classes, young (2 weeks of age), adult (5 to 21 weeks of age) and old (52 to 104 weeks of age), for identification of hepatic age-related DEMs. Age-related DEMs were identified independently in females and males using a *t* test with an FDR of <5% and requiring a fold change difference of at least 1.5. When comparing young to adult, 159 male DEMs and 131 female DEMs (Fig. 5a) were identified as age-related DEMs. Among these, 115 were in common, suggesting similar miRNA expression patterns in males and females. Indeed, 88% of the female DEMs were also differentially expressed in males. There were also large numbers of DEMs when comparing young to old rats, 161 in males and 148 in females, with 125 (>77%) in common (Fig. 5b). This indicates that there are large differences in the liver miRNA profiles between young rats and adult and old rats, and much smaller differences between adult and old rats. Comparing the miRNA liver expression profiles between adult and old rats identified only 17 DEMs in males and 14 in females, of which 6 were in common (Fig. 5c). The miRNAs in each comparison are shown in Additional file 3.

As noted above, *k*-means cluster analysis identified 38 miRNAs (clusters 2 and 3 in Fig. 2) that showed a 2-week-specific expression pattern, i.e., there was high expression in the livers of 2-week-old rats but low/undetectable expression at later ages (expression values are shown in Additional file 4). The expression of one of these miRNAs (miR-376b-3p) through the life span is shown in Fig. 4. In males, the expression of miR-376b-3p at 2 weeks of age was >320-fold higher than the average expression at all other ages, while in females it was >150-fold higher. In males, 35 of the 38 miRNAs were expressed at >10-fold greater levels in 2-week-old animals than at all subsequent ages. In females, 28 of the 38 miRNAs were expressed at >10-fold greater levels in 2-week-old animals than at all subsequent ages. Furthermore, male expression was higher than female expression for all except one (miR-675*) of the two-week-specific miRNAs. On average, the expression of these 38 hepatic miRNAs was 1.9-fold higher in males than in females at 2 weeks of age. The genes for 31 of the 38 miRNAs are located on rat chromosome 6 (see Additional file 2 and Additional file 4).

### Verification of microarray data by qPCR

Four DEMs that showed significant sex and/or age differences by microarray analysis (miR-29b, miR-96, miR-154*, and miR-34a) were selected for verification by Taqman qPCR assays. Pearson correlation coefficients for the measurements by the two methods for the four miRNAs ranged from 0.835 to 0.966 (shown in Fig. 6), suggesting good agreement between the two methods of expression analysis. Measurement of miR-29b expression by microarray showed female-biased expression at 15, 21, 52, and 78 weeks of age. Significant (*p* < 0.05) sex difference was confirmed by qPCR for miR-29b only at 52 weeks of age (female > male, Fig. 6a). Microarray data for miR-96 showed male-biased expression at 21, 52, and 78 weeks of age. Taqman qPCR results confirmed this male-biased miR-96 expression at 78 weeks of age and additionally at 104 weeks of age (Fig. 6b). By microarray measurement, miR-154* showed 156- and 383-fold increased expression at 2 weeks of age compared to the average expression at all subsequent ages in females and males, respectively. This large 2-week-specific expression difference was verified in the qPCR data, which showed 2-week expression differences of 1212-fold and 4169-fold in females and males (Fig. 6c), respectively. The larger fold changes determined by qPCR may be due to the larger dynamic range of the qPCR assay. In addition, the significant male-biased expression at 2 weeks of age was confirmed by qPCR. Significant age-related increases in miR-34a expression (7.1- and 13.2-fold maximum age difference in females and males, respectively; microarray data) were also confirmed by qPCR, which showed significant (*p* < 0.05) 7.2- and 8.7-fold differences in expression across the life span in females and males, respectively (Fig. 6d). Thus, there was good correlation between the expression levels determined by microarray and qPCR, although expression differences of <2-fold were not always confirmed.

### Potential pathways and functions of DEMs

Potential mRNA targets of the DEMs were identified using Ingenuity Pathway Analysis software, restricting the analysis to experimentally validated interactions retrieved from TarBase, miRecords, and curated from the peer-reviewed literature. The predicted mRNA targets were then examined for over-representation in biological pathways curated in Ingenuity Pathway Analysis.

### 2-week-old-specific hepatic miRNA expression

38 miRNAs were identified in the *k*-means cluster analysis as being expressed in the livers of pre-weaning 2-week-old rats and expressed at reduced levels or not detectable levels at all subsequent ages (Fig. 2, clusters 2 and 3; Additional file 4). Table 1 shows the 38 miRNAs in this 2-week-specific class along with the mRNA targets and associated pathways/functions where the mRNAs were significantly enriched. A relatively small number of mRNA targets (Capn8, Cntn4, Onecut1, Tagln, and Vim) was identified using rat-specific data from IPA and no significant pathways were identified. Human-specific miRNA-mRNA association data were, therefore, included in the analysis and this expanded the mRNA targets by 5, giving a total of 10 mRNA targets for the 38 miRNAs. Only 6 of the miRNAs had associated mRNAs, and these mRNAs were significantly represented in the molecular functions: cellular development, cellular growth and maintenance, cellular growth and proliferation, cell morphology, and cell-to-cell signaling. Notably, 3 of the mRNAs (BCL6, PRDM1, and XBP1), all targets of miR-127, were associated with the hematological system.

**Table 1:** mRNA targets and pathways/functions associated with differentially expressed miRNAs

miRNA class	miRNAs in class^a^	# of miRNAs in class	Predicted mRNA targets^b^	# of mRNA targets	Potentially affected pathways/functions^c^
2-week-specific expression	*miR-127*, miR-127*, *miR-136*, miR-136*, miR-154, miR-154*, miR-299, miR-300-3p, miR-322*, miR-329, miR-337, miR-337*, miR-341, miR-3563-3p, miR-369-5p, miR-376a, miR-376b-3p, miR-376b-5p, miR-376c, miR-379, miR-381, *miR-382*, miR-409-5p, miR-410, miR-411, miR-411*, *miR-431*, *miR-434*, miR-434*, miR-455, miR-483, miR-487b, *miR-495*, miR-541, miR-542-3p, miR-542-5p, miR-582, miR-675*	38	BCL6, CAPN8, CNTN4, ONECUT1, PRDM1, RTL1, SEPT3, TAGLN, VIM, XBP1	10	*Molecular functions:* Cellular development Cellular growth and maintenance Cellular growth and proliferation Cell morphology Cell to cell signaling *Physiological systems:* Hematological system
Sex difference in adults	*miR-100, miR-107, miR-125b-5p*, miR-139-3p, miR-144, miR-151, *miR-152*, miR-191*, *miR-200b, miR-203, miR-21, miR-221, miR-27b, miR-290, miR-29b, miR-326,* miR-339-3p, miR-340-3p, *miR-34b*, miR-374, miR-425, miR-466b-2*, miR-505*, miR-532-3p, miR-598-3p, miR-652, miR-802, *miR-96, miR-99a*, miR-99a*	30	Acvr2a, Adamts1, Adcy6, Adora1, Adora2b, Akap6, Apaf1, Aqp5, Arc, Axin2, B3galt4, Bace1, Bak1, Bcl2, Bcl2l11, Bmf, Bmpr2, Bnip3l, Btg2, Capn8, Casp6, Casp7, Cbln2, Cbx7, Ccnd1, Ccne1, Cd276, Cdc25a, Cdca4, Cdk6, Cdkn1a, Cdkn1b, Cdkn2a, Cebpg, Celsr2, Cntn4, Col1a1, Col1a2, Col3a1, Col4a1, Col5a3, Crkl, Cyp1a1, Cyp1b1, Ddit4, Dicer1, Dio3, Dll1, Dnmt1, Dnmt3a, Dnmt3b, Dusp2, E2f5, Elmo2, Erbb2, Erbb3, Errfi1, Esr1, Fadd, Fam3c, Fbn1, Fgf16, Fgfr3, Fos, Golph3, Gpr160, H3f3b, Hdac4, Hipk3, Htr1b, Icam1, Icos, Id1, Id2, Id3, Igf1r, Igfbp3, Il1rn, Irs1, Jag1, Kcnj16, Kit, Klf15, Lamc1, Madd, Map2k1, Mapk1, Mcl1, Met, Mgst1, Mitf, Mmp13, Mmp9, Myc, Myrip, Nfib, Notch1, Notch2, Nr1i2, Nr4a1, Odc1, Pdcd4, Peli1, Pex7, Phb, Pigr, Pik3r1, Plag1, Plk1, Pparg, Ppp2r2a, Pten, Ptprd, Rere, Runx1, Rxra, Ryk, Serpinb5, Sgpl1, Slc16a10, Smad3, Smad4, Smad5, Smo, Socs3, Sod2, Sox5, Sp1, Sparc, Spry2, Srm, Ssr3, St14, St18, Stx1a, Syt4, Tagln, Tdg, Tgfb3, Timp3, Tp53, Tpm1, Tspan8, Ube2i, Ugt2b, Vegfa, Vsnl1, Wisp2, Yy1	149	*Molecular functions:* Cellular growth and proliferation Cell death and survival Cellular development Gene expression Cell cycle *Toxicological functions:* Liver necrosis Liver proliferation Liver fibrosis Hepatocellular carcinoma Liver hyperplasia
Sex difference in old	*miR-125b-5p, miR-152, miR-183, miR-29b, miR-31*, miR-31*, miR-340-5p, miR-374, *miR-494, miR-92a, miR-96, miR-99a*, miR-99a*	13	Acvr2a, Adamts1, Adcy6, Aqp5, B3galt4, Bace1, Bak1, Bcl2l11, Bmf, Bmpr2, Btrc, Casp6, Casp7, Casr, Cbln2, Cbx7, Cd276, Cdc25a, Cdk6, Cdkn2a, Cebpg, Celsr2, Col1a1, Col1a2, Col3a1, Col4a1, Col5a3, Cyp1a1, Dicer1, Dio3, Dnmt1, Dnmt3a, Dnmt3b, Dusp2, Erbb2, Erbb3, Fbn1, Fgf16, Fgfr3, Gpr160, H3f3b, Hdac4, Hif1a, Hipk3, Hmox1, Htr1b, Id1, Id2, Id3, Igf1r, Igfbp3, Il1rn, Irs1, Lamc1, Map2k4, Mcl1, Mitf, Myrip, Nr1i2, Pdgfb, Pigr, Pik3r1, Plk1, Ppp2r2a, Pten, Ryk, Scn3a, Sgpl1, Smo, Sp1, Sparc, St18, Tdg, Tgfb3, Tp53, Tpm1, Tspan8, Ube2i, Ugt2b, Vsnl1, Yy1	81	*Molecular functions:* Cellular death and survival Cellular development Cellular growth and proliferation Gene expression Cell cycle *Toxicological functions:* Liver necrosis
Difference between adults and old (male)	*miR-125b-5p, miR-146b, miR-200b*, miR-203, *miR-221*, miR-29a, *miR-29b*, miR-29c, miR-324-5p, *miR-34a, miR-375*, miR-455*, miR-466b-2*, miR-598-3p, miR-802, *miR-99a*, miR-99a*	17	Acvr2a, Adamts1, Adipor2, Axin2, B3galt4, Bace1, Bak1, Bcl2, Bcl2l11, Blmh, Bmf, Bnip3l, Brca1, C1qbp, Casp6, Casp7, Cbln2, Cbx7, Ccna2, Ccnd1, Cd276, Cdc25a, Cdk6, Cdkn1b, Cdkn2a, Cebpg, Cfh, Col1a1, Col1a2, Col3a1, Col4a1, Col5a3, Cxcr4, Cyp1a1, Ddit4, Dicer1, Dio3, Dll1, Dnmt3a, Dnmt3b, Dusp2, E2f5, Elmo2, Erbb2, Erbb3, Errfi1, Esr1, Fadd, Fbn1, Fgf16, Fgfr3, Fos, Gpr160, H3f3b, Hdac4, Icam1, Id1, Id2, Id3, Igf1r, Igfbp3, Il1rn, Irak2, Jag1, Jak2, Kif22, Kit, Lamc1, Ltb, Map2k1, Mcl1, Met, Mettl7a, Mmp16, Mtpn, Myc, Notch1, Notch2, Pa2g4, Pigr, Pik3r1, Plekha4, Plk1, Ppp2r2a, Ptprd, Rere, Sdcbp2, Sgpl1, Smo, Socs3, Sod2, Sp1, Sparc, St18, Stat1, Tagln, Tdg, Tgfb3, Timeless, Tp53, Tpm1, Tspan8, Ube2i, Ugt2b, Usp1, Vegfa, Wisp2, Ywhaz, Yy1	109	*Molecular functions:* Cellular death and survival Cellular growth and proliferation Gene expression Cell cycle Cellular development *Toxicological functions:* Liver necrosis/cell death Hepatocellular carcinoma Liver hyperplasia Liver proliferation Liver fibrosis
Difference between adults and old (female)	*miR-1, miR-133b, miR-182*, miR-183, *miR-18a*, miR-206, miR-29a, *miR-29b*, miR-29c*, miR-34a*, *miR-375*, miR-466b, miR-466b-2*, *miR-96*	14	Acvr2a, Adar, Adcy6, Adipor2, Aqp5, Arcn1, Arf3, Arf4, Arhgap29, Atp6v1b2, Axin2, Axl, Bace1, Bcl2, Bcl2l11, Bdnf, Blcap, Btrc, C1qbp, Calm3, Cand1, Cap1, Ccnd1, Cd276, Cdc42, Cdk6, Cdk9, Celsr2, Clcn3, Cnn3, Col1a1, Col1a2, Col3a1, Col4a1, Col5a3, Ctgf, Ddx5, Dll1, Dnmt3a, Dnmt3b, Dusp2, E2f5, Esr1, Fbln2, Fbn1, Fstl1, Gja1, H3f3b, Hand2, Hdac4, Htr1b, Igf1, Igf1r, Irs1, Jag1, Jak2, Kcnj2, Klf15, Lamc1, Lasp1, Lin7c, Lztfl1, Map2k1, Mcl1, Met, Mitf, Mmd, Mtpn, Myc, Myrip, Nfatc4, Notch1, Notch2, Notch3, Oat, Pdcd4, Pik3r1, Pim1, Pitx3, Pola1, Pom121, Rabl2a, RGD1359334, Rhoa, Rnf138, Runx2, Ryk, Sdc4, Serpinb5, Sh2d4a, Slc25a30, Sp1, Sparc, Srxn1, Tac1, Tagln, Tagln2, Tdg, Tdp1, Tgfb3, Thbs1, Timp3, Tns4, Tpm1, Trappc3, Trim2, Tspan4, Uhmk1, Usp1, Utrn, Vegfa, Wisp2, Xpo6, Ywhaz, Yy1	115	*Molecular functions:* Cellular death and survival Cellular growth and proliferation Gene expression Cellular development Cell cycle *Toxicological functions:* Liver necrosis/cell death Liver proliferation

^a^miRNAs in italics have known mRNA targets in the IPA database

^b^Derived from rat-specific miRNA-mRNA associations except for the 2-week-specific miRNA class, which included both rat- and human-specific miRNA-mRNA associations in IPA

^c^From IPA pathway/function analysis with criteria of at least three mRNAs per pathway/function and a Fisher’s exact test *p* value < 10^−3^

### Sex different miRNA expression in the liver of adult and old rats

As shown in Fig. 3, 30 hepatic miRNAs were found to be differentially expressed between the sexes in adults (5–21 weeks of age), and 13 were found to be differentially expressed in old rats (52–104 weeks of age). Table 1 shows the sex different miRNAs in these two age groups, as well as the target mRNAs and the significantly affected pathways/functions. Target mRNA information was available for at least half of the miRNAs for these two groups (15/30 for the adults and 9/13 for old), resulting in 149 mRNA targets in the adults and 81 mRNA targets in the old group. The top five most significant molecular functions affected by the mRNA targets of the sexually dimorphic miRNAs were identical between the two age groups: cell death and survival, cellular growth and proliferation, cellular development, gene expression, and cell cycle. These mRNA targets also appeared to be involved in liver toxicology functions, with more significant effects in the adults (significantly affected functions included liver necrosis, liver proliferation, liver fibrosis, hepatocellular carcinoma, and liver hyperplasia) than old rats (significantly affected functions included only liver necrosis).

### Hepatic miRNA expression differences between adult and old rats

As shown in Fig. 5c and Additional file 3, there were 25 miRNAs whose hepatic expression differed between adult (5–21 weeks of age) and old (52–104 weeks of age) rats, with 17 different between adult males and old males, and 14 different between adult females and old females. The expression of 6 of these miRNAs differed between adult and old rats in both sexes; the expression of 11 differed only in male rats, and the expression of 8 differed only in female rats. Table 1 shows these age different miRNAs in males and females, along with the target mRNAs and significantly affected pathways/functions. Target mRNA information was available for about half of the miRNAs (8/17 for the males and 8/14 for the females), resulting in 109 mRNA targets for males and 115 mRNA targets for females. The top five molecular functions were identical for both the males and the females (cell death and survival, cellular growth and proliferation, gene expression, cellular development, and cell cycle), and were the same as found for the sex differences in adults and sex differences in old rats. The liver toxicological function differences between adult and old rats were similar in each sex, with liver necrosis and liver proliferation functions found in both sexes. There were additional significant liver toxicological function differences between adult and old males that were not seen between adult and old females, and these included hepatocellular carcinoma, liver hyperplasia, and liver fibrosis. The similarity in pathway/function differences between adult and old in both sexes may be the result of the large overlap in mRNA targets; 47 mRNA targets were in common between the sexes.

### Association of hepatic miRNA expression with liver histopathology

Liver histology sections were examined for standard pathological findings at 52, 78, and 104 weeks of age. Sex bias in the incidence of three types of lesions was observed (Table 2). These included basophilic focus, hyperplasia of the bile duct, and vacuolization in hepatocyte cytoplasm. Examples of the histological presentation of these lesions are shown in Fig. 7. Female-biased incidence of basophilic foci was evident at 78 weeks (3 of 8 females showed the lesion compared to 0 of 8 males) and 104 weeks (12 of 16 females compared to 0 of 14 males). As is typical for histopathological findings of basophilic foci, no severity score was provided [53]. Basophilic foci are considered pre-neoplastic lesions; however, no neoplasms were observed in any female animals. One neoplastic lesion (classified as hepatocellular adenoma), however, was observed in a male animal at 104 weeks of age. This is consistent with the slightly higher incidence rate for hepatocellular adenoma in normal males (2.3%) compared to females (0.44%) at two years of age, according to National Toxicology Program historical control animal data for F344 rats [54]. Male-biased incidence of hyperplasia of the bile duct was observed at 52 weeks of age (8 of 10 males compared to 0 of 10 females), 78 weeks of age (7 of 8 males compared to 2 of 8 females), and 104 weeks of age (10 of 14 males compared to 2 of 16 females). Severity scores among animals showing the lesion ranged from minimal to mild in all cases. Male-specific incidence of hepatocyte cytoplasm vacuolization was observed at 52 weeks of age (5 of 10 males), 78 weeks of age (2 of 8 males), and 104 weeks of age (1 of 14 males) compared to zero incidence in females of the same ages. Average severity scores for this lesion also ranged from minimal to mild, with one case of moderate (78 weeks) and one case of marked (104 weeks). All findings were considered common for F344 rats of these ages.

**Table 2:** Sex differences in liver histopathology

Lesion		52 weeks of age				78 weeks of age				104 weeks of age			
		Female		Male		Female		Male		Female		Male	
	Sex bias	Incidence	Avg. severity score^b^	Incidence	Avg. severity score^b^	Incidence	Avg. severity score^b^	Incidence	Avg. severity score^b^	Incidence	Avg. severity score^b^	Incidence	Avg. severity score^b^
Basophilic focus^a^	Female	0/10	–	0/10	–	3/8	–	0/8	–	12/16	–	0/14	–
Hyperplasia, bile duct	Male	0/10	–	8/10	1.6	2/8	1.5	7/8	1.4	2/16	2	10/14	1.9
Vacuolization, cytoplasm, hepatocyte	Male	0/10	–	5/10	1.4	0/8	–	2/8	2.5	0/16	–	1/14	4

^a^Basophilic loci scored as present or absent, without a severity score [53]

^b^Average among those animals showing the lesion

As can be seen in Table 2, there is a substantial sex difference in the prevalence of basophilic foci in the livers of 78- and 104-week-old rats, with females showing this histopathology while males did not. The histopathology was not observed in males or females of 52 weeks of age, or in males of 78 or 104 weeks of age. Basophilic foci were not found (0/12 males and 0/12 females at 6–7 week of age and 0/24 males and 0/24 females at 17–18 weeks of age) in contemporaneous studies at NCTR using F344 rats from the same colony and living in the same environmental conditions at NCTR [47]. Using the expression level from 5 to 21 weeks of age (adults) as a baseline where no liver histopathology was observed, the fold change in expression of each miRNA was calculated at 52, 78, and 104 weeks of age for each sex separately. miRNAs were excluded if the expression value was less than 0.3 (60^th^ percentile of expression level distribution; approximately 40% of expression values were undetectable) in all age groups. Significantly changed expression was defined as *p* < 0.05 and fold change ≥ 1.5 between the adult age group and 52, 78, or 104 weeks of age groups for each sex independently. A total of 43 miRNAs met these criteria and are shown in Additional file 5. The expression of two miRNAs was associated with the presence and absence of basophilic foci, miR-18a, and miR-451 (Table 3). The expression of miR-18a was reduced relative to the 5–21-week-old baseline group when basophilic foci were present, i.e., significantly decreased expression was seen in the livers of 78 and 104 week old (1.7-fold decrease) females and not in any of the males or 52-week-old female livers. The expression of miR-451 was significantly increased relative to the 5–21-week-old baseline group when basophilic foci were present, i.e., increased expression was seen in the livers of 78 (1.8-fold increase) and 104-week-old (1.5 fold increase) females and not in any of the males or 52-week-old female livers.

**Table 3:** Correlation of miRNA expression with hepatic basophilic foci^a^

Feature	52 week F	78 week F	104 week F	52 week M	78 week M	104 week M
Basophilic focus^b^	No (0%)^c^	Yes (38%)	Yes (75%)	No (0%)	No (0%)	No (0%)
miR-451	NS	1.8	1.5	NS	NS	NS
miR-18a	NS	0.6	0.6	NS	NS	NS

*F* female, *M* male, *NS* not significant

^a^Relative expression values are the expression at the indicated ages relative to the average expression from 5 to 21 weeks of age for each sex independently

^b^From Table 2

^c^Incidence from Table 2

Table 2 also shows a substantial sex difference in the prevalence of bile duct hyperplasia, with >80% of males from 52 to 104 weeks of age having this histopathology. It was not seen in females of 52 weeks of age, and occurred in <20% of females of 78 and 104 weeks of age. This lesion was not observed (0/12 males and 0/12 females at 6–7 week of age and 0/24 males and 0/24 females at 17–18 weeks of age) in contemporaneous studies at NCTR using F344 rats from the same colony and living in the same environmental conditions at NCTR [47]. Using the same analysis described above, the expression of 2 miRNAs was found to associate with the presence and absence of liver bile duct hyperplasia, miR-99a and miR-203 (Table 4). The expression of both miRNAs was significantly increased in the livers of males from 52 to 104 weeks of age relative to the expression in the livers of the 5–21-week-old baseline group (1.6- to 2.5-fold). The expression of the 2 miRNAs was not significantly changed in the livers of females of 52 to 104 weeks of age.

**Table 4:** Correlation of miRNA expression with hepatic bile duct hyperplasia^a^

Feature	52 week F	78 week F	104 week F	52 week M	78 week M	104 week M
Hyperplasia, bile duct^b^	No (0%)^c^	Minimal (25%)	Minimal (13%)	Yes (80%)	Yes (88%)	Yes (71%)
miR-99a	NS	NS	NS	2.0	1.7	1.6
miR-203	NS	NS	NS	2.5	2.2	2.0

*F* female, *M* male, *NS* not significant

^a^Relative expression values are the expression at the indicated ages relative to the average expression from 5 to 21 weeks of age for each sex independently.

^b^From Table 2

^c^Incidence from Table 2

## Discussion

### General trends in the expression of liver miRNAs during the rat lifespan

Age and sex differences were evaluated independently to identify liver miRNA expression changes during the rat life span. The 214 DEMs included 212 miRNAs which showed significant age differences and 65 miRNAs exhibiting significant sex differences. PCA (Fig. 1) and *k*-means cluster analysis (Fig. 2) provided visualization of the variability of individuals within age/sex groups, between groups, and among the individual miRNAs. Three general observations followed from these analyses. First, age plays a much larger role than sex in these hepatic miRNA expression changes. Notable difference was observed between 2-week-old animals and all subsequent age groups in the PCA (Fig. 1), and aside from a small sex difference observed between 2-week-old males and females, no large-scale patterns of sex difference were observed. Second, the notable separation in the PCA between 2-week-old animals and all subsequent age groups was found to be caused by 38 two-week-specific miRNAs (Fig. 2 and Additional file 4). Third, aside from miRNAs showing 2-week-specific expression, liver miRNAs exhibited relatively stable expression across both sex and age, with >70% of the miRNAs having coefficient of variation <50% (Fig. 2). These differential expression analyses and data visualization methods revealed key insights into the data that helped focus pathway analysis of the DEMs.

### Sex-biased miRNAs linked to key liver pathways

Sex differences in miRNA expression were evaluated at each age group (Fig. 3). The distribution of sex-biased miRNAs identified in the liver showed a similar pattern as the sex-biased miRNAs observed previously in kidney tissues [34] from the same animal study. Both kidney and liver showed significant sex-biased miRNA expression at 2 weeks of age and then again at ages of 15 weeks and older, with a gap of no observed sex-biased miRNAs between 5 and 6 weeks of age. Previously reported liver mRNA expression study revealed a similar pattern of sex-biased regulation, where 5- and 6-week-old rats showed lower numbers of sex-biased mRNAs compared to neighboring age groups, including the 8–21-week-old groups [15]. This suggests either that sex-biased gene expression change is not as crucial during the pubertal developmental stage compared to other life stages, or that the more subtle or fine-tuned miRNA regulation may not be involved in the hormonally controlled sexually dimorphic expression changes that do exist during this developmental time frame. Among the 65 sex-biased DEMs, only 6 (miR-92a, miR-221, miR-374, miR-505*, miR-532-3p, and miR-652) are encoded on the X-chromosome. Four of these miRNAs exhibited female-biased expression, and two exhibited male biased expression (miR-92a and miR-221). miR-221 showed male-biased expression at 8, 15, and 21 weeks of age, exhibiting a 14-fold difference in expression (21 weeks) compared to females. This was the second highest fold change difference among all sex-biased miRNAs, with only male biased miR-183 having a greater sex-biased expression (17-fold). As there was no consistency in the sex-biased expression of the miRNAs, it is not clear what role X-chromosome inactivation may play in the control of sex-biased miRNA expression. No miRNAs are encoded on the rodent Y-chromosome, and only two miRNAs are Y-chromosome-encoded in humans [55].

The miRNAs with the most consistent female-biased expression were miR-29b (Fig. 3) and miR-152 (Fig. 3 and Fig. 4) with both showing an approximate 2-fold higher expression in females than males at 15, 21, 52, and 78 weeks of age. miR-29b is a key repressor of renal and pulmonary fibrosis [56, 57] and has recently been shown to inhibit hepatic fibrogenesis by directly targeting PIK3R1 and AKT3 in hepatic stellate cells [58]. The role of the miR-29 family, including miR-29b, in fibrosis disease has been recently reviewed and additional targets appear to be collagens [59]. miR-152 is dysregulated in rat models of NAFLD [60] and has been characterized to act as a tumor suppressor [61]. Thus, these sexually dimorphic miRNAs may play a role in differential susceptibility to liver pathology and disease.

miRNAs showing the most male-biased expression included miR-125b-5p, miR-99a*, miR-99a, miR-96, miR-221, and miR-183 (Fig. 3). miR-125b-5p and miR-99a showed the most consistent male-biased expression with sex differences observed at 15, 21, 52, and 78 weeks of age and fold changes ranging from 1.5 to 2.3. miR-99a* exhibited similar significant male-biased expression of 1.7- to 2.3-fold at 15, 52, and 78 weeks of age. miR-125b-5p and miR-99a are suggested to be co-expressed in humans from chromosome 21 and play a role in vincristine resistance of leukemia cells [62]. miR-221 and miR-96 showed relatively larger sex differences over the middle portion of the life span, with miR-221 expressed at 3-to 14.3-fold higher levels in males than females from 8 to 21 weeks of age, and miR-96 expressed at 2.2 to 7.2 higher levels in males than females from 21 to 78 weeks of age. miR-125b-5p and miR-221 play a role in glioma carcinogenesis and have been recently proposed as prognostic markers in assessing glioma tumors [63]. The expression of miR-183 exhibited the largest identified sex difference with 17.2-fold higher expression in males compared to females at 104 weeks of age. miR-183 and miR-96 are co-transcribed from the miR-183-96-182 cluster found on chromosome 4 in rats and chromosome 7 in humans [64], suggesting that post-transcriptional processing may independently control the expression of these miRNAs. MiR-183 has been shown to function in a pro-oncogenic manner by suppressing apoptosis and promoting proliferation in esophageal cancer cells [65].

The expression of 30 miRNAs was sexually dimorphic in the liver of adult (5 to 21 weeks of age) rats and there is evidence that they are involved in the regulation of 149 mRNAs (Table 1). The top five molecular functions associated with these mRNAs (cellular growth and proliferation, cell death and survival, cellular development, gene expression, and cell cycle) represent important pathways whose disruption or modification may be expected to contribute to disease susceptibility. In fact, by focusing on toxicological functions using the IPA database, these mRNAs appear to be involved in multiple processes related to liver disease, including necrosis, proliferation, fibrosis, hepatocellular carcinoma, and hyperplasia. Thus, this analysis suggests the sexually dimorphic expression of miRNAs described in this study may be reflective of sex differences in liver physiology and disease susceptibility.

In older rats from 52 to 104 weeks of age, the hepatic expression of 13 miRNAs was found to be sexually dimorphic, with evidence that they are involved in the regulation of 81 mRNAs (Table 1). The top five molecular functions associated with these mRNAs are identical to those described above for the sexually dimorphic mRNA targets in adults. Despite only seven sexually dimorphic miRNAs being in common between the adult and the old rats (miR-125b-5p, miR-152, miR-29b, miR-374, miR-96, miR-99a*, and miR-99a), 91% (74/81) of the mRNA targets in the old rats were also mRNA targets in the adult rats. This suggests that multiple miRNAs target the same mRNAs resulting in a similar impact on molecular functions. The toxicological functions of these mRNAs, however, were more limited in old rats compared to the adults. Only one significant toxicological function was associated with the 81 mRNA targets, liver necrosis. Thus, sexually dimorphic hepatic miRNA expression in old rats is mostly a subset of that in adults. The reduction in sexually dimorphic miRNA expression seen in this study is consistent with the finding of increased similarity of the male and female hepatic mRNA expression pattern at the oldest ages [15].

### Two-week-specific miRNAs linked to Dlk1-Dio3 cluster

A prominent feature of the *k*-means cluster (Fig. 2 and Additional file 4) is the high expression of 38 miRNAs in clusters 2 and 3 at 2 weeks of age with no to little expression at subsequent ages. A review of the miRNA annotations which included chromosome locations revealed a notable trend. Of the 38 miRNAs showing 2-week-specific expression, 31 of them (81%) were encoded on rat chromosome 6. Closer investigation revealed that these chromosome 6-encoded miRNAs were members of the delta-like 1 homolog-deiodinase, iodothyronine 3 (Dlk1-Dio3) miRNA cluster. The conserved DLK1-DIO3 miRNA cluster (located on 14q32 in humans) is the largest miRNA cluster in the human genome and includes 54 encoded miRNAs [66]. The Dlk1-Dio3 genomic region is an imprinted region containing two differentially methylated regions which control its regulation [67]. A subset of miRNAs within the Dlk1-Dio3 miRNA cluster, called the miR-379/410 cluster (containing 38 miRNAs), has been shown to be essential for neonatal liver energy homeostasis (hepatic glucose and lipid metabolism) in mice [68]. miR-379, miR-410, and 16 other miRNAs from this subcluster were among the 2-week-specific miRNAs in our study and were present in *k*-means clusters 2 or 3. Their association with neonatal energy homeostasis is consistent with their expression at 2 weeks of age, when animals are transitioning from maternal energy sources to independent feeding. miRNA members of the Dlk1-Dio3 cluster have also been shown to be activated in embryonic stem cells [69] and positively correlate with stem cell pluripotency and proliferation [70]. In a mouse model of myocardial infarction, 29 miRNAs originating from the Dlk1-Dio3 cluster were activated in cardiomyocytes after induced tissue damage [71], suggesting their role in tissue remodeling efforts to replace damaged cardiac tissue. Taken together, these findings suggest a role for Dlk1-Dio3 cluster miRNAs in liver proliferation, differentiation, and energy homeostasis at 2 weeks of age, consistent with the potentially affected pathways/functions (e.g., cellular growth and proliferation, cellular development, cellular growth and maintenance) identified from possible mRNA targets of these miRNAs (Table 1).

In addition, the miRNAs expressed predominantly at 2 weeks of age have predicted mRNA targets that impact the hematological system (Table 1). These three target mRNAs (PRDM1, BCL6, and XBP1) are important transcription factors and regulators of B and T cell differentiation, with all three encoded proteins interacting with each other [72, 73]. This is consistent with the mid-gestation to early juvenile expression of hematopoiesis in the liver, which ends by postnatal day 35 [74]. Thus, the high level expression of these miRNAs at 2 weeks of age and the dramatically reduced levels by 5 weeks of age correlates with the hematopoietic potential of the liver.

### miRNA hepatic expression differences between adult and old rats

The expression of a small number of hepatic miRNAs significantly differed between adult (5–21 weeks of age) and old (52–104 weeks of age) rats, 17 in males and 14 in females. The expression of six (miR-29a, miR-29b, miR-29c, miR-34a, miR-375, and miR-466b-2*) was altered in both males and females (Table 1). There were 109 potential target mRNAs for the 17 male miRNAs and 115 for the 14 female miRNAs, with 77 (~42%) found in both males and females. This high degree of overlap of the mRNA targets resulted in the prediction of similar molecular function changes as both males and females matured from adults to old age. Three of the five most significantly affected functions predicted to be affected included cellular death and survival, cellular growth and proliferation, and cell cycle. The miR-29 family and miR-34a, whose expression was altered in both older males and older females, are relatively well-studied miRNAs, and their roles in cell proliferation and apoptosis are established [75, 76]. In addition, the miR-29 family members appear to play a significant role in the development of liver fibrosis, possibly through the regulation of collagen expression [59]. Thus, miRNA control of important functions of liver maintenance appears to be altered in aged males and females, and may contribute to the age-associated susceptibility to liver disease, including fibrosis [77].

### miRNA expression associates with histopathology

Tables 3 and 4 show the expression of specific miRNAs associated with the presence or absence of specific liver histopathology. Increased expression of miR-451 and decreased expression of miR-18a were associated with the presence of hepatic basophilic loci, which are considered pre-neoplastic lesions [53]. miR-451 has recently been suggested as a prognostic biomarker of hepatocellular carcinoma (HCC), with decreased expression in HCC tissues correlating with advanced stage, metastasis, and worse disease-free or overall survival [78]. In vitro studies demonstrated inhibition of cell growth, possibly through direct suppression of the NF-κB pathway [79] suggesting that the increased expression associated with the presence of basophilic foci, observed in the present study, may be a compensatory response to the pre-neoplastic lesion. In addition, increased serum levels of miR-451 were recently found in patients with NAFLD [80]. Altered expression of miR-18a has also been implicated in the development of HCC through modulation of the estrogen receptor (ER) alpha protein [81] with increased expression promoting the downregulation of the ER alpha. Because the estrogen pathway appears to be protective for HCC [82], this may be a key factor in explaining the increased susceptibility of males to HCC. The decreased expression of miR-18a found to be associated with basophilic foci in the present study may, again, be a compensatory response to the pre-neoplastic basophilic foci.

Increased expression of both miR-99a and miR-203 was associated with the presence of hepatic bile duct hyperplasia, a common aging lesion [83]. miR-99a appears to be a tumor suppressor in the liver [84] that possibly acts through inhibition of the RNA-induced silencing complex, Ago2 [85]. Several studies suggest that miR-203 also acts as a tumor suppressor in the liver [86–88] by inhibition of liver cell proliferation. Altered expression of miRNA-203 has also been found to be associated with alcoholic steatohepatitis [89]. Thus, even though the exact role of these miRNAs in the development of hepatic basophilic foci and bile duct hyperplasia is not known, as described above, these miRNAs have been shown in a variety of in vitro, in vivo, and bioinformatics studies to have a fundamental impact on liver cell biology. Additional targeted studies will be needed to evaluate these miRNAs as possible biomarkers for these liver lesions and their possible roles in development of age- and sex-dependent susceptibility to liver pathology.

### Association of miRNA and mRNA expression

miRNAs have been shown to regulate protein expression by a diverse set of mechanisms, some of which destabilize mRNA targets [19]. Combining miRNA and mRNA expression levels may allow a fuller understanding of the influence of miRNAs on mRNA expression in the rat liver. mRNA expression in the livers of rats from this same study, measured by microarrays, has been previously published [15] and are available in the gene expression omnibus database (GSE21335). Preliminary analysis of the expression of mRNA targets listed in Table 1 shows that 226 of the 464 mRNA targets (49%) were expressed at detectable levels. Of these detectable mRNA targets, the expression of an average of 26% of mRNAs in each class was significantly (*p* < 0.05) changed with an inverse relationship to the miRNA expression change; that is, when the miRNA was significantly upregulated, the target mRNA was significantly downregulated, or vice versa. Thus, the stability of a substantial fraction of the detectable target mRNAs appears to be directly affected by the miRNAs listed in Table 1.

The relationship between miRNAs and their targets, however, is complex and not completely understood [90]. For example, multiple miRNAs can affect a single mRNA, and a single miRNA can affect multiple mRNAs. Since the miRNAs may control protein expression by mRNA degradation and/or inhibition of translation, there may not be a direct inverse relationship between miRNA levels and target mRNA levels. In addition, there may be other factors that affect expression, such as DNA methylation, transcription factors, and histone modifications. Also, the current state of knowledge regarding miRNA-mRNA relationships in the liver is incomplete. The relative lack of sensitivity of the older microarray technology used to measure mRNA levels [15] may be responsible for the low detectability of the predicted target mRNAs. However, better quantitative and sensitive methods, such as RNA-Seq, may allow more accurate evaluation of the correlation of miRNA expression with target mRNA expression. Additional confirmatory computational and experimental analyses, including protein quantitation, will be required to fully understand the role of miRNAs and other gene regulatory mechanisms in the control of hepatic gene expression.

## Conclusions

As a key first step in understanding epigenetic mechanisms that may influence sex- and age-related susceptibilities to adverse liver events, genome-wide characterization of liver miRNA expression profiles in both sexes during the rat life span was carried out. miRNAs exhibited largely stable expression between sexes and across the rat life span with the exception of a conspicuous pattern of 38 miRNAs showing 2-week-specific expression. This pattern at 2 weeks of age was explained in large part by co-localization of 31 miRNAs to the Dlk1-Dio3 cluster on chromosome 6. This cluster is putatively associated with early developmental proliferation and differentiation, and postnatal liver energy homeostasis, which is consistent with the developmental events occurring in immature animals at 2 weeks of age. Pathway analysis of sex-biased miRNAs in adult and old rats identified sexually dimorphic molecular functions and toxicological functions that may reflect sex differences in liver physiology and disease susceptibility. The expression of specific miRNAs (miR-451, miR-18a, miR-99a, and miR-203) was found to associate with sexually dimorphic histopathology findings of basophilic foci and bile duct hyperplasia. As rats mature from adults to old-age, miRNAs involved in cell death, cell proliferation, and cell cycle (miR-29 family and miR-34a) were found to change expression. In addition, miR-29 family members may be involved in susceptibility to fibrosis. Thus, the expression of miRNAs involved in the control of important liver functions was shown to change during the rat life span and between the sexes, and may contribute to the age- and sex-associated susceptibility to liver disease.

## Additional files

Additional file 1: 212 differentially expressed miRNAs by age for each sex. The age contrast with differential expression is given along with fold-ratio, ANOVA FDR, degrees of freedom (df), ANOVA F statistic, and *p* values for both males and females. (XLSX 232 kb)
Additional file 2: 214 miRNAs differentially expressed by age and/or sex. Each miRNA is shown as differentially expressed by age, sex, or both age and sex, along with the *k*-means cluster number from Fig. 2 and chromosome mapping position. (XLSX 20 kb)
Additional file 3: Age-related differences in miRNA expression. Identification of miRNAs in Venn diagrams in Fig. 5. (XLSX 26 kb)
Additional file 4: 38 miRNAs with 2-week-old-specific expression. Identification of 2-week-old-specific miRNAs, along with expression level in each rat liver sample, *k*-means cluster number (from Fig. 2), differential expression by age and/or sex, and chromosome mapping position. (XLSX 34 kb)
Additional file 5: 43 significantly differentially expressed miRNAs between 52, 78, and 104 weeks of age males and females compared to 5–21 weeks of age baseline group. Presence or absence of histopathological findings is given with the relative fold change in expression of the miRNAs. (XLSX 10 kb)

## Abbreviations

DEMs: Differentially expressed miRNAs
DILI: Drug-induced liver injury
Dlk1-Dio3: Delta-like 1 homolog-deiodinase, iodothyronine 3
ER: Estrogen receptor
FDR: False discovery rate
FE: Feature Extraction
HCC: Hepatocellular carcinoma
IPA: Ingenuity Pathway Analysis
miRNAs: microRNAs
NAFLD: Non-alcoholic fatty liver disease
NCTR: National Center for Toxicological Research
NS: Not significant
PC: Principal component
PCA: Principal component analysis
qPCR: Quantitative PCR
RINs: RNA integrity numbers
TGS: Total gene signal
